# Appraising protein conformational changes by resampling time-resolved serial x-ray crystallography data

**DOI:** 10.1063/4.0000258

**Published:** 2024-07-24

**Authors:** Adams Vallejos, Gergely Katona, Richard Neutze

**Affiliations:** Department of Chemistry and Molecular Biology, University of Gothenburg, Box 462, 40530 Gothenburg, Sweden

## Abstract

With the development of serial crystallography at both x-ray free electron laser and synchrotron radiation sources, time-resolved x-ray crystallography is increasingly being applied to study conformational changes in macromolecules. A successful time-resolved serial crystallography study requires the growth of microcrystals, a mechanism for synchronized and homogeneous excitation of the reaction of interest within microcrystals, and tools for structural interpretation. Here, we utilize time-resolved serial femtosecond crystallography data collected from microcrystals of bacteriorhodopsin to compare results from partial occupancy structural refinement and refinement against extrapolated data. We illustrate the domain wherein the amplitude of refined conformational changes is inversely proportional to the activated state occupancy. We illustrate how resampling strategies allow coordinate uncertainty to be estimated and demonstrate that these two approaches to structural refinement agree within coordinate errors. We illustrate how singular value decomposition of a set of difference Fourier electron density maps calculated from resampled data can minimize phase bias in these maps, and we quantify residual densities for transient water molecules by analyzing difference Fourier and Polder omit maps from resampled data. We suggest that these tools may assist others in judging the confidence with which observed electron density differences may be interpreted as functionally important conformational changes.

## INTRODUCTION

Structural biology is a tremendously successful field of research that has contributed valuable insights into how the three-dimensional structures of proteins, DNA, and RNA govern their function. Time-resolved macromolecular x-ray crystallography aims to resolve time-dependent changes in the position of atoms and the structural rearrangements that represent the trajectories of biological reactions.^1–3^ There are several ingredients that must be fulfilled for a time-resolved x-ray diffraction (TR-XRD) experiment to be successful. First and foremost, the protein target of interest must yield well diffracting crystals and its x-ray structure should have been determined. There must also be a means of initiating the biochemical reaction that can be synchronized throughout a crystal, which is at least as rapid as the scientific question of interest. Moreover, for x-ray diffraction data to be collected, the conformational changes initiated within a crystal should not be so large that they cause the crystal lattice to disorder and x-ray diffraction to be lost. Finally, there must be accepted tools for structural analysis so that time-dependent changes in diffraction intensities are interpreted in terms of protein structural changes in a quantitative manner.

Synchrotron radiation was critical as the field of time-resolved macromolecular crystallography developed. The first demonstrations of time-dependent changes in electron density at room temperature achieved a time-resolution of several minutes.^4,5^ Time-resolved Laue diffraction was extended into the millisecond to nanosecond domain^6,7^ by observing light-induced electron density changes in crystals of carboxy myoglobin^8^ and photoactive yellow protein.^9,10^ The scope of these studies was later expanded to other biological systems^11,12^ and was applied to the study of light-induced changes in integral membrane protiens,^13^ and the time-resolution attained using synchrotron radiation was extended into the picosecond domain.^14^

Since the first demonstrations of serial femtosecond x-ray crystallography^15–18^ (SFX) using an x-ray free electron laser^19^ (XFEL), the collection of x-ray diffraction data at room temperature has enjoyed a revival.^20^ Critically, a proof of principle demonstration of time-resolved SFX (TR-SFX) using microcrystals of photoactive yellow protein as a model system^21^ illustrated the power of collecting and merging x-ray diffraction data from thousands of microcrystals, each exposed to only a single XFEL pulse. This demonstration was very important since it showed that, despite the shortcomings of each and every individual measurement from randomly oriented crystals of variable size using an XFEL pulse that fluctuated shot-to-shot, it was still possible to extract very high-quality difference Fourier electron density maps from TR-SFX data. This sparked a new approach to time-resolved crystallography, which has allowed the field to rapidly expand.^2^ Some noteworthy achievements include the characterization of light driven conformational changes in retinal proteins^22–27^ including the primary G protein-coupled receptor of mammalian vision;^27^ other isomerization reactions within proteins;^28,29^ studies of charge separation reactions of photosynthesis^30^ and the coupled oxygen evolving reaction;^31–35^ and photodissociation reactions,^36^ including the first example of protein conformational dynamics in the single-photon excitation domain,^37^ which provided an important link between the previously separate domains of time-resolved crystallography^2^ and ultrafast spectroscopy.^38^

In this special issue of *Structural Dynamics*, we discuss the challenge of interpreting time-dependent changes in x-ray diffraction intensities in terms of protein conformational changes, an area in which the Moffat group has made many substantial contributions.^8–10,39,40^ More specifically, we consider accepted tools of structural analysis and reflect upon how the resampling of serial x-ray crystallography data may be utilized to accurately represent variation in observed and modeled structural changes. To illustrate this approach, we utilize time-resolved serial femtosecond x-ray crystallography (TR-SFX) data previously reported for structural changes within the light-driven proton pump bacteriorhodopsin (bR).^22^ Specifically, the absorption of a photon by retinal molecule that is covalently bound to Lys216 of helix G, induces its photoisomerization from an all-*trans* to a 13-*cis* conformation. This rearrangement of retinal initiates a cascade of conformational changes throughout the protein that lead to the transport of a proton against a transmembrane proton concentration gradient.

We first benchmark how the magnitude of conformational changes captured in the TR-SFX data varies as the occupancy is varied, illustrating that there is a domain over which the magnitude of Cα displacements is inversely proportional to the modeled crystallographic occupancy for both partial occupancy refinement and refinement against extrapolated x-ray diffraction data. Resampling procedures are used to estimate coordinate uncertainties, from which we conclude that the Cα displacements recovered using both partial occupancy structural refinement and structural refinement against extrapolated data agree within experimental errors. Singular value decomposition of difference electron density maps calculated using resampled data is used to demonstrate that the choice of the resting state model does not unduly bias the isomorphous difference Fourier electron density map calculations. Finally, we illustrate how resampling can also yield estimates of the uncertainty in the amplitude of difference Fourier electron density peaks, and Polder omit map peaks, associated with selected water molecules. We suggest that these versatile resampling approaches may be useful when a sequence of time-resolved serial crystallography data are interpreted in terms of protein conformational changes.

## METHODS

All analyses were developed using TR-SFX data collected at SACLA from photoactivated microcrystals of bR, as previously reported.^22^ More specifically, the stream files from this sequence of experiments are publicly available and were recovered from the CXI data bank^41^ (CXIDB ID 53). Stream files provide the values of partial x-ray intensities recorded frame by frame, which equates to microcrystal by microcrystal in the context of serial x-ray crystallography data. Fourteen stream files, corresponding to x-ray diffraction data of a resting state along with thirteen logarithmically spaced time delays, were processed to reproduce the refined structures using a pipeline developed in-house. Programs from the CrystFEL^42^ suite (v0.10.1) were used to reduce the data by first resolving the indexing ambiguity of space group P6_3_ using AMBIGATOR and scaled and merged using PARTIALATOR, while figures of merit were monitored using CHECK_HKL and COMPARE_HKL. The CCP4^43^ suite (v8.0.015) was used to convert the reflections to MTZ format using F2MTZ, and structure factor amplitudes were obtained by TRUNCATE and combined by CAD.

An in-house PYTHON (v3.7.12) pipeline was written to generate fixed-size (10 000 selected diffraction patterns) bootstrapped subsets of observations with replacement. One hundred separate, but not independent, datasets for the resting state and for thirteen photo-activated datasets were generated, amounting to 1400 resampled datasets in total. For each of these resampled datasets, a structure was resolved and refined using the following protocol. Partial occupancy structural refinement was performed by modeling two alternate conformations A and B of all atoms with complementary occupancies between 1% and 99%, where atomic coordinates and B-factors of conformation B (the activated state) were free to change during refinement and conformation A (the resting state) was held fixed. Extrapolation of merged crystallographic structure factor amplitudes was performed using the program XTRAPOL8^44^ (v1.2.0) with the “fgenick” method and occupancies ranging from 1% to 99% using the phases and amplitudes of the resting state. Five cycles of real-space, reciprocal-space, individual B-factor, and structural refinement were performed using PHENIX.REFINE from the PHENIX^45^ suite (v1.19.2). PDB entry 5B6V was used as a starting model, with the retinal modified to adopt a 13-*cis* conformation for the photoactivated state (*c.w.* all-*trans* retinal for the resting state).

Isomorphous *F*_obs_(light) − *F*_obs_(dark) difference Fourier electron density maps were calculated using the program PHENIX.FOBS_MINUS_FOBS_MAP,^45^ using the phases of the corresponding refined resting state structure. As such, when calculating 100 difference Fourier electron density maps from 100 resampled light and dark datasets, the phases were slightly different for each calculation due to slight differences between resting state structures refined against resampled data. Polder OMIT maps were determined using PHENIX.POLDER, and the intensities were integrated within a sphere of radius 0.8 Å centered on selected water atoms using a modified version of a procedure previously described.^46^ The principal (first) components of singular value decomposition^30,40,47^ (SVD) of each set of 100 isomorphous *F*_obs_(light) − *F*_obs_(dark) difference Fourier electron density maps (one for each time delay) were calculated using an in-house PYTHON code, where matrix *A* of isomorphous difference maps is decomposed as *A* = *USV^T^*, where *A* is a *n* × *m* matrix with *m* equal to the number of resampled difference maps, and *n* elements in each map, *U* is a unitary matrix of *n* × *n* elements, S is a *n* × *m* diagonal matrix of singular values arranged in decreasing order, and *V* is a *m* × *m* matrix of right singular vectors.

## RESULTS

The fraction of molecules activated onto a reaction trajectory is called the crystallographic occupancy of the activated state, which we give the symbol *f*. As a first step in the structural analysis of any TR-XRD study, the isomorphous difference Fourier electron density map is almost always calculated and presented. Henderson and Moffat showed that the difference Fourier method is far more sensitive to small changes in electron density than any other type of electron density map^39^ and this tool has remained the gold-standard for TR-XRD ever since. An isomorphous difference Fourier electron density map is calculated as a Fourier transform (*FT*) of the differences in experimental structure factor amplitudes (called *F*_obs_) using crystallographic phases [Φ(reference)] calculated from the refined reference state (resting or dark) conformation,

$$FT\;[(F_{\text{obs}}(\text{activated})-F_{\text{obs}}(\text{reference}))\times \text{exp}(i\Phi (\text{reference}))].$$ (1)

Since no information on the activated state structure is incorporated into this calculation, the map is free from bias toward the activated state, but it is not free of model bias should the difference amplitudes contain substantial systematic or random errors. Moreover, the nature of features observed in a difference Fourier map should not depend upon the crystallographic occupancy, although the signal-to-noise ratio of a difference Fourier electron density map improves as the crystallographic occupancy increases. Difference Fourier analysis, however, requires that the unit cell parameters of both the activated and resting states are almost identical (isomorphous) and on rare occasions the process of initiating a reaction within crystals leads to the loss of crystal isomorphism. Positive difference electron density features in a difference Fourier map indicate that electron density that was not present in the resting conformation has arisen from the reaction of interest, whereas negative difference electron density features indicate that electron density has been lost as the reaction proceeds. Schemes for improving difference Fourier electron density maps have been developed based upon Bayesian analysis,^44,48^ and singular value decomposition can also be used to improve signal to noise in a sequence of difference Fourier maps.^30,40^ For visualization purpose, it may be useful to project changes in electron density from a difference Fourier map analysis onto the protein sequence.^46^

### Amplitude of atomic motions vs crystallographic occupancy

Determining the crystallographic occupancy is a critical step in modeling the structural changes that are captured within the crystallographic data. When processing serial crystallography data, it will not be true that the crystallographic occupancy and conformational distribution within all microcrystals are the same. However, after merging x-ray diffraction data, it is usually most convenient and robust that one value, *f*, for the crystallographic occupancy is chosen for the activated state conformation. Despite this problem being central to structural analysis, there are no universally accepted standards for determining the crystallographic occupancy. Several different approaches for determining *f* have been used and include varying the occupancy until the B-factors of selected atoms match those of neighboring atoms,^22^ to searching for correlations in the difference Fourier maps and optimizing certain parameters.^44^ In some cases, the occupancy is allowed to vary from one time-point to another,^22,30^ whereas others have sought to fix the crystallographic occupancy in a systematic way over a sequence of time-dependent data.^23,37,49,50^

Two main strategies are used in TR-XRD for modeling of conformational changes. Partial occupancy refinement proceeds by having two (or on rare occasions more) conformations modeled in a pdb file. In this approach, the conformation of the resting (e.g., dark) structure is given an occupancy of 1 − *f* and the resting conformation is usually (but not always) held fixed. Conversely, the structure of the activated (light) conformation is given an occupancy of *f*, and both the atomic coordinates and crystallographic B-factors of this conformation are allowed to vary during structural refinement. Sometimes a limited number of protein atoms are allowed to change their positions during structural refinement, and these regions are usually selected based upon the presence of significant electron density features associated with these atoms or regions in the *F*^obs^ − *F*^obs^ difference Fourier electron density maps.^22,51^ On other occasions, the atomic coordinates of the entire structure are allowed to move. Another variant of partial occupancy refinement is to utilize ensemble refinement,^52^ which combines molecular dynamics simulations with structural refinement against x-ray diffraction data so as to better capture functionally important dynamical motions of proteins.^52^ In that approach, ensembles of protein structures are found, which together fit the x-ray diffraction data by better accounting for anisotropic coordinate displacements. However, there are many limitations to structural modeling including the risks of overfitting data as well as the lack of reproducibility, and as such, subjective decisions taken by the scientists may influence the extent or nature of subtle structural changes in the final model.

**FIG. 1.**
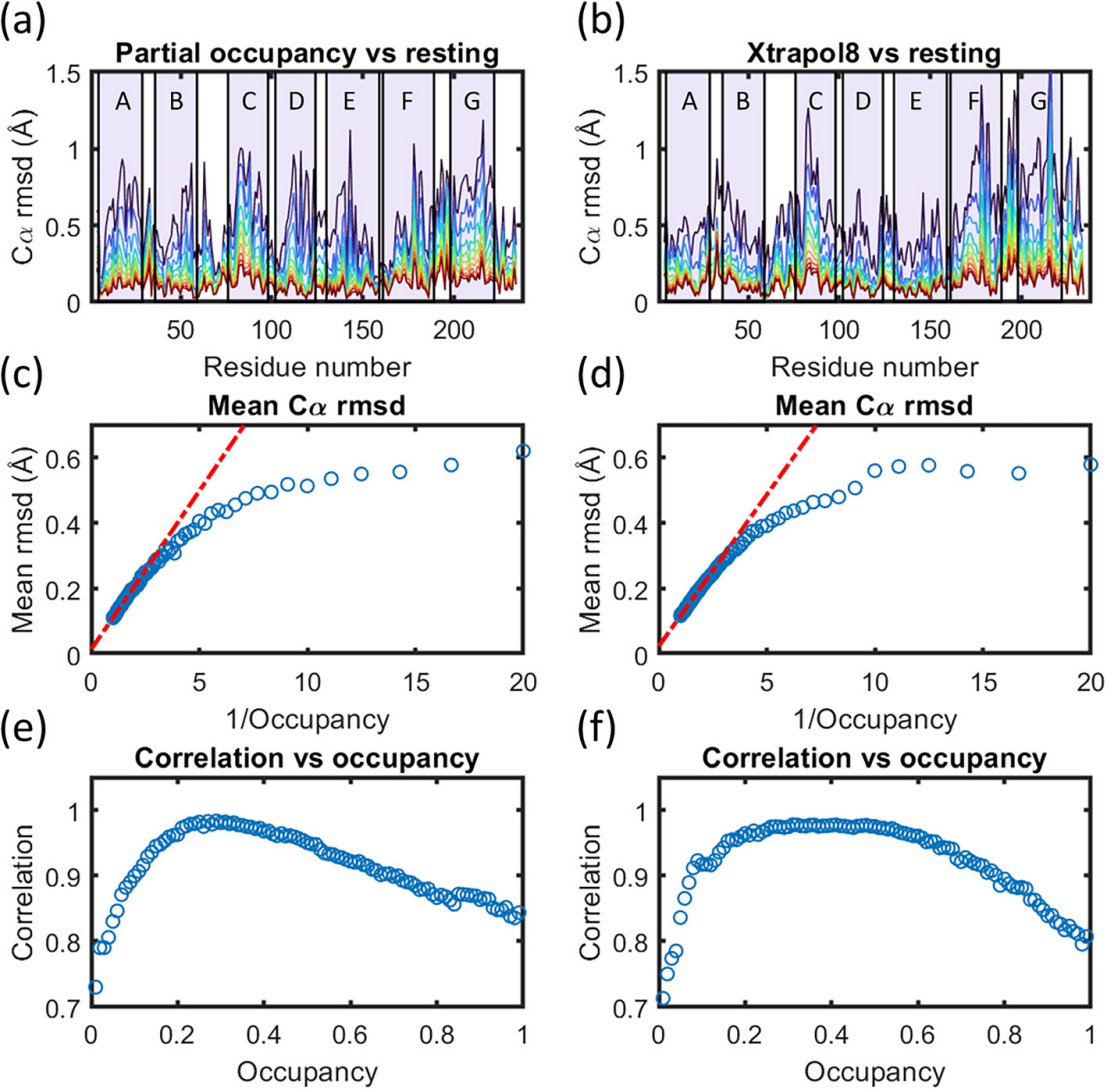
Influence of crystallographic occupancy on the amplitude of motions during structural refinement. (a) Cα-atom root mean square displacements (rmsd) from the fixed resting structure following structural refinement using partial occupancy. The results for an evenly spaced 10% change in occupancy *f* from 9% to 99% are overlaid. Transmembrane helices are indicated in light purple and are labeled A to G. (b) Cα-atom rmsd from the fixed resting structure following structural refinement using extrapolated data calculated using the package XTRAPOL8.^44^ The results for an evenly spaced 10% change in occupancy from 9% to 99% are overlaid. In panels a and b, the amplitude of Cα-atom rmsd is colored blue to red as the occupancy increases. (c) Mean rmsd of Cα-atom as a function of the crystallographic occupancy when using partial occupancy structural refinement. (d) Mean rmsd of Cα-atom as a function of the crystallographic occupancy when using structural refinement against extrapolated data. (e) Pearson correlation coefficient of Cα-atom rmsd curves for any given occupancy vs the average Cα-atom rmsd curve from the entire set of structural refinements using partial occupancy structural refinement. A maximum correlation is observed in the domain 27% to 29%. (f) Pearson correlation coefficient of Cα-atom rmsd curves for any given occupancy vs the average Cα-atom rmsd curve from the entire set of structural refinements using structural refinement against extrapolated data. All structural refinements were performed using PHENIX,^45^ and both atomic coordinates and B-factors for the activated state were allowed to change during refinement. During partial occupancy refinement, the atomic coordinates and B-factors of the resting conformation were held fixed, with a complementary occupancy (1 − *f* ).

In Fig. 1, we illustrate how the amplitude of motions modeled for bR against TR-SFX data^22^ using the time-delay Δ*t* = 1.725 ms vary as the crystallographic occupancy is varied from 1% to 100%. More specifically, the root mean square deviation (rmsd) of Cα atoms for the light (activated) conformation, the model in which all coordinates and B-factors of the protein's atoms are allowed to vary during refinement, is calculated relative to the coordinates of Cα atoms of the dark (reference) conformation, which was held fixed during structural refinement. The output from this calculation is illustrated in Fig. 1(a) for occupancy values from 9% to 99% in steps of 10%, illustrating that the amplitude of conformational changes varies with occupancy. Within the domain 25% ≤ *f* ≤ 100%, there is a linear relationship between 1/*f* and the mean amplitude of conformational changes [Fig. 1(c)]. If the modeled occupancy is low (i.e., 1/*f*  ≥ 4), the magnitude of the modeled conformational changes continues to increase as the occupancy decreases, but this magnitude no longer varies linearly with 1/*f*. Using the same set of structures derived using partial occupancy structural refinement, Fig. 1(e) illustrates the Pearson correlation coefficient between the rmsd of Cα atoms and the mean rmsd for the entire set. A useful feature of the Pearson correlation coefficient is that it is independent of the amplitude of the motion, and therefore, this plot illustrates how closely the motions captured by structural refinement for any given occupancy agree with those captured by the entire set. Interestingly, this analysis shows a maximum at 27% ≤ *f* ≤ 29%, which is slightly lower than that estimated in Nango *et al.* ( *f* = 34% for these data-set). These results illustrate how the nature of modeled conformational changes is largely independent of the chosen occupancy, but the amplitude of these motions depends critically upon the choice of *f*.

Another popular approach to modeling conformational changes is to refine against extrapolated data. In this approach, it is assumed that it is possible to subtract the structure factor amplitudes of the reference conformation from the activated conformation and thereby create a hypothetical full-occupancy dataset for the activated conformation. Structural refinement then proceeds at full occupancy against these extrapolated data. There are several variations of this approach, but the most straightforward applies the following formula:

$$F_{\text{obs}}(\text{extrapolated})=1/f\times F_{\text{obs}}(\text{activated})+(1-1/f)\times F_{\text{obs}}(\text{reference}).$$ (2)

When considering structural refinement against extrapolated data, we use this formula as it is implemented using the program XTRAPOL8.^44^

As was observed using partial occupancy refinement, the amplitude of the rmsd changes of Cα positions varies as the occupancy is varied from 9% to 99% [Fig. 1(b)]. Moreover, the mean amplitude of Cα displacements across the protein again varies linearly with 1/*f* over the domain 25% ≤ *f* ≤ 99% [Fig. 1(d)]. When refining against extrapolated data, however, there is a much broader plateau for the region where the Pearson correlation coefficient on rmsd of Cα atoms is most strongly correlated with the mean of the entire set [Fig. 1(f)]. It is also noteworthy that the maximum correlation arises 30% ≤ f ≤ 50%, which is higher than that obtained using partial occupancy refinement and is also outside of the domain for the crystallographic occupancy estimated using the tools of XTRAPOL8 against these datasets,^44^ which suggested that the occupancy was 18% ≤ *f* ≤ 23%.

Comparing the rmsd on Cα atoms shown in Figs. 1(a) and 1(b), it appears that structural refinement against extrapolated data shows more distinctive domains where the Cα atoms depart from the reference conformation, whereas this contrast is less obvious when using partial occupancy refinement. Quality control metrics, such as R-factor and R-free, also fall within an accepted range when using partial occupancy refinement, where we obtain R-factor = 18.6% and R-free = 18.7% when performing structural refinement using an occupancy of 27%. By contrast, in this case we obtain R-factor = 26.5% and R-free = 26.8% when refining against extrapolated data again using an occupancy of 27%. It has long been acknowledged that the rational for structural refinement against extrapolated data is an approximation based upon idealized assumptions^44,53^ and frequently the crystallographic metrics are significantly worse. The reason that R-factors are significantly worse when using extrapolated data is that the procedure of extrapolation necessarily exaggerates experimental errors in the data.

### Estimating coordinate errors using resampled data

The quality of isomorphous difference Fourier electron density maps is frequently regarded as the best measure of the success of a TR-XRD study.^2,39^ Conversely, if paired negative and positive difference electron density features represent atomic motions, how confident can we be that these motions have been captured by structural refinement, and could regions of a protein that do not show significant features in the difference Fourier map nevertheless display protein rearrangements that are functionally significant? In this context, an often-underappreciated consideration in TR-XRD studies is how best to estimate coordinate errors for the refined structure of the activated state.

Considerable empirical understanding has emerged for how to estimate coordinate errors in single crystal x-ray crystallography, and these underpin a proposal for a simplified formula for estimating coordinate errors^54^ in the case of 100% crystallographic occupancy,

$$\sigma_{\text{free}}^{2}=0.65\cdot \frac{N_{a}}{N_{o}}\cdot R_{\text{free}}^{2}\cdot d_{\text{min}}^{2}\cdot C^{3/2},$$ (3)

where $\sqrt{\sigma_{\text{free}}^{2}}$ estimates of the overall coordinate error in the structure; C is the completeness, $R_{\text{free}}$ is recovered from crystallographic refinement, $d_{\text{min}}$ is the maximum resolution of the data, $N_{a}$ is the number of atoms included in refinement, and $N_{o}$ is the number of independent observations. This formula suggests a mean coordinate error for the resting state SFX structure of bR^22^ of 0.10 Å. However, it is unclear from this formalism how the partial occupancy of the conformation of interest impacts upon coordinate error estimates, nor how the very high multiplicity of serial crystallography data should impact on this analysis.

In an idealized case, coordinate errors would be estimated from the spread of coordinates from a set of structures after refinement against multiple independent x-ray diffraction datasets. In practice, it is seldom the case that the multiplicity of serial crystallography data supports this approach. Therefore, resampling methods can be applied, which generate multiple datasets for which the resampled diffraction patterns overlap to some degree, and coordinate errors have been estimated from the spread of coordinates after structural refinement against resampled datasets.^30,55,56^ Alternatively, the noise in crystallographic data can be simulated, and the spread of coordinates after refinement against simulated data can be used to estimate coordinate errors.^35^

**FIG. 2.**
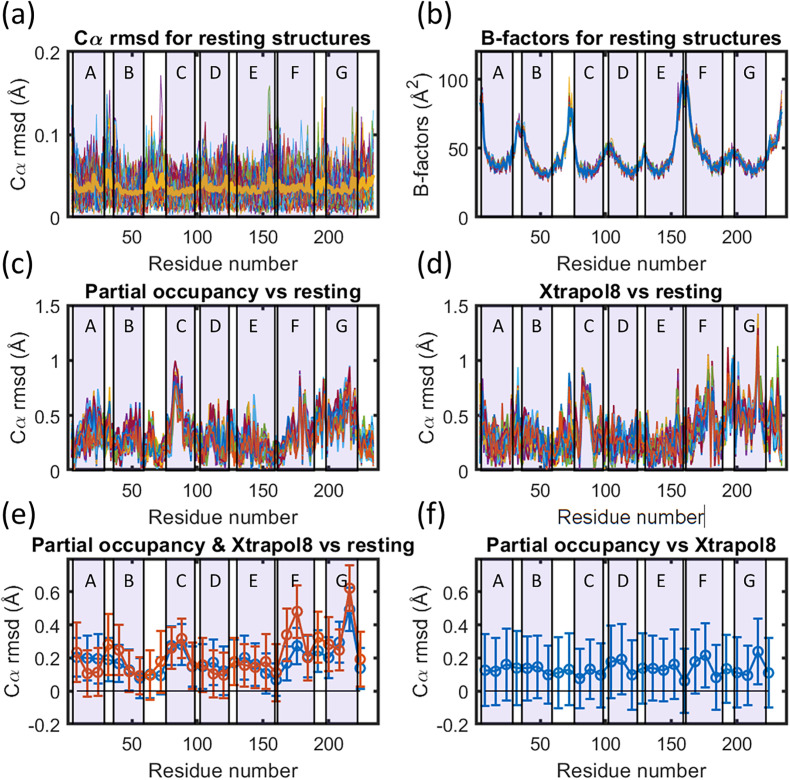
Comparison of the results of structural refinement with partial occupancy and against extrapolated data. (a) Estimation of coordinate errors on Cα-atoms using bootstrap resampling of the resting state conformation. Thin lines show the rmsd of Cα-atoms from the average Cα-atom coordinate from a set of 100 structural refinements against bootstrapped resampled data. Overlaid upon, this set (thick mustard line) is the average rmsd of Cα-atoms from the mean of these 100 structures. Larger uncertainty is visible for loop regions. (b) Crystallographic B-factors of all Cα-atoms extracted from the same set of 100 structures refined using bootstrap resampling of the resting state conformation. Larger B-factors are visible for loop regions. (c) Cα-atom rmsd from the resting structure following partial occupancy structural refinement against light activated data corresponding to the time-delay Δ*t* = 1.725 ms, using an occupancy of 27%. The results from 100 structural refinements against bootstrap resampled data are shown, with the activated state coordinates and B-factors allowed to vary and the resting state conformation held fixed. (d) Cα-atom rmsd from the fixed resting structure following structural refinement against extrapolated data generated using XTRAPOL8. In this case, light activated data corresponding to the time-delay Δ*t* = 1.725 ms were resampled, and a parallel resampling of the dark dataset was used to extrapolate to full occupancy assuming, a light-activated state occupancy of 27%. The results from 100 structural refinements are shown. (e) Error bars and rmsd of Cα-atoms extracted from panels c and d for partial occupancy refinement (blue) and refinement against extrapolated data (red). (f) Separation of the mean Cα-atom coordinates recovered from partial occupancy refinement from the mean Cα-atom coordinates recovered from refinement against extrapolated data, and their corresponding error bars. This establishes that the two refinement methods are in agreement within error bars. Transmembrane helices are indicated in light purple and are labeled A to G.

Figure 2(a) illustrates the root mean square deviation (rmsd) of Cα-coordinates when using bootstrapping resampling to generate 100 datasets, each of which contain 10 000 indexed diffraction patterns. These data were sampled from a 2.0 Å resolution SFX resting structure of bR^22^, which contained almost 250 000 diffraction patterns. Since 100 sets of 10 000 resampled diffraction patterns equate to 10^6^ diffraction patterns, these resampled data are not fully independent and each image will be selected four times on average. This quasi-independent resampling gave a mean rmsd on Cα atoms of 0.04 Å and the mean rmsd on all atoms of 0.14 Å, the latter value being slightly larger than the value of 0.10 Å estimated using Eq. (3). The observed distribution of coordinate variations correlates with the distribution of B-factors [Fig. 2(b)] in line with Cruickshank's DPIU model.^57,58^ Crystallographic B-factors are incorporated into structural refinement with the explicit purpose of allowing for variations in the refined atomic positions to influence the predicted x-ray diffraction amplitudes. Using the definition that B = 8π^2^⟨u^2^⟩, the mean B-factor value of 41 Å^2^ observed for the resting structure of bR gives ⟨u⟩ = 0.7 Å. This value is a factor of five larger than that recovered for coordinate error estimates, illustrating that the mean fluctuations about an average atomic position are much larger than the sphere of confidence within which one can refine the coordinates of any atomic position.

Estimates of coordinate errors for an activated conformation may be expected to be higher when the activated state occupancy is low. To examine this point, we applied a parallel approach to that used above by performing partial occupancy refinement of the activated conformation against TR-SFX data recorded from bR microcrystals for the time-delay Δ*t* = 1.725 ms. For the sake of consistency, we applied an occupancy of 27% for the activated state, which was the value having the highest correlation with the mean rmsd from 99 refinements with occupancy from 1% to 99% [Fig. 1(e)]. One hundred structural refinements with partial occupancy were made for the coordinates and B-factors of the activated state against resampled data using 27% occupancy, during which the resting state conformation was held fixed to the coordinates and B-factors of pdb entry 5B6V with an occupancy of 63%. All 100 rmsd curves calculated for Cα positions are shown in Fig. 2(c) for the activate conformation relative to the resting conformation. Since only 13 648 diffraction patterns were indexed for this time-delay,^22^ each resampled dataset overlapped to a much larger extent with the other resampled datasets than when resampling from the resting conformation dataset. Despite this lack of independence, this procedure yielded a mean photo-activated coordinate error on Cα atoms of 0.09 Å [Fig. 2(e)] and a mean coordinate error on all atoms of the photo-activated state of 0.12 Å. This procedure yielded a larger coordinate error estimate for the partial occupancy conformations for Cα-positions, but a very similar coordinate error estimate on all atoms. It is possible that this uncertainty value is underestimated somewhat due to the lower number of diffraction patterns from which to resample data.

Finally, coordinate errors associated with structural refinement against extrapolated data were also examined. The same 100 resampled datasets associated with Δt = 1.725 ms were used as light datasets, and the same resampled dark datasets that were used to generate Fig. 2(a) were used as reference data. Equation (2) was then applied to extrapolate data to recover one hundred quasi-full occupancy datasets for the photoactivated state assuming 27% occupancy (*f* = 0.27) as implemented in XTRAPOL8.^44^ One hundred structural refinements were then performed against these extrapolated data, with one full-occupancy activated state conformation allowed to vary coordinates and B-factors. All 100 rmsd curves calculated for Cα positions are shown in Fig. 2(d) for the activate conformation relative to the resting conformation. Applying the same procedures as above to determinate coordinate errors, we recover a mean coordinate error on Cα atoms of 0.11 Å and the mean rmsd on all atoms of 0.17 Å for the 13-*cis* photoactivated conformation. These coordinate error values for structural refinement against extrapolated data are slightly larger than coordinate error estimates made for the dark conformation and those for the photoactivated conformation using partial occupancy refinement.

**FIG. 3.**
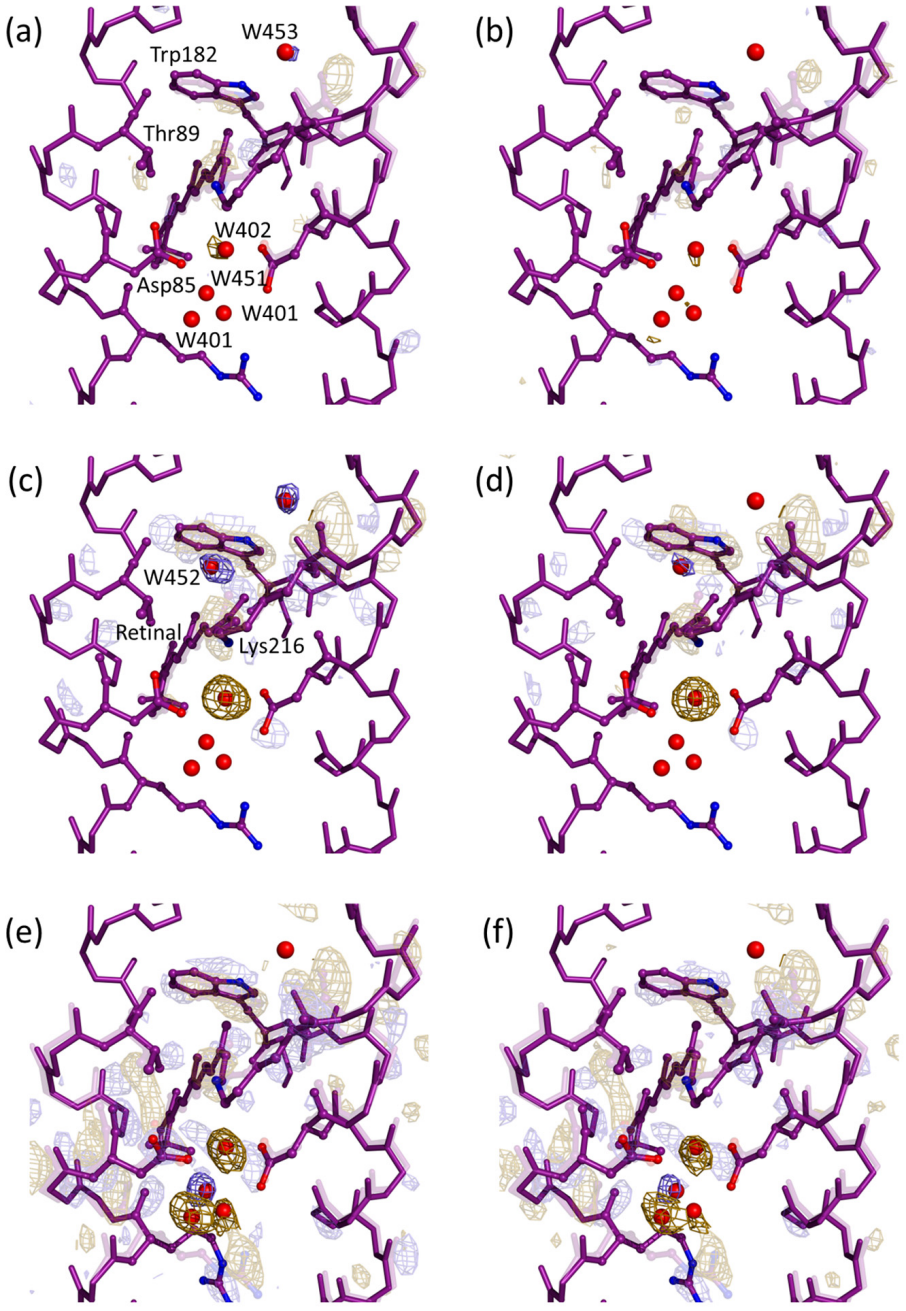
Comparison of isomorphous *F*_obs_(light) − *F*_obs_(dark) difference Fourier electron density maps calculated from resampled data and merged using singular value decomposition (SVD), and the corresponding difference Fourier map calculated from the light-activated and resting states without resampling. (a) The principal SVD component determined from 100 difference Fourier maps recovered by resampling data for Δt = 16 ns and the dark conformation. (b) Control difference Fourier map recovered for Δt = 16 ns without resampling. (c) The principal SVD component determined from 100 difference Fourier maps recovered by resampling data for Δt = 760 ns and the dark conformation. (d) Control difference Fourier map recovered for Δt = 760 ns without resampling. (e) The principal SVD component determined from 100 difference Fourier maps recovered by resampling data for Δt = 1.725 ms and the dark conformation. (f) Control difference Fourier map recovered for Δt = 1.725 ms without resampling. All data were extracted from stream files provided by Nango *et al.*^22^ Blue corresponds to positive difference electron density, whereas gold corresponds to negative difference electron density. Highly transparent mesh is contoured at ±3.5σ. Nontransparent mesh is contoured around selected water molecules and is contoured at ±3.0σ, where σ is the root mean square electron density of the map.

With these tools in place, we are able to compare the results from two separate approaches of structural refinement of the photoactivated state, and to examine if the structural conclusions are consistent with each other given coordinate errors. As illustrated in Figs. 2(c) and 2(d), there is a high degree of similarity in the nature of the rmsd displacements of Cα atoms. Most importantly, whereas structural refinement did not predetermine which regions were allowed to move, it is apparent that the largest motions are associated with helix C (largest displacements are between residues 82 to 88), with significant displacements of Cα atoms associated with the helical turn from 178 to 182 near the center of helix F, where Trp182 sits directly to the cytoplasm of the retinal and comes into steric conflict with its C20 methyl group after photoisomerization [Fig. 3(a)]; and throughout helix G, where the largest displacement is associated with Lys216 to which retinal is covalently bound [Fig. 3(c)]. However, there is no significant perturbation of the cytoplasmic E–F loop, which is believed to tilt outwards as Trp182 is displaced toward the cytoplasm^25,59^ and this motion is hinged by Pro186. As was previously noted, this lack of movement is presumably due to crystal packing interactions between residues 165 and 166 of the E–F loop and residues 232 and 234 of the C-terminus restricting the allowed motions.^22^ Very extended motions were suggested from the electron diffraction structure of a bR triple-mutant, which was constitutively open,^59^ and were modeled with modest occupancy against x-ray diffraction data collected using time-resolved serial synchrotron crystallography^25^ with a time-delay of several milliseconds after photoactivation. Numerous other x-ray crystallography intermediate trapping studies, however, failed to capture these motions,^60^ and the addition of reagents to optimize the flow of the LCP microjet may have fortuitously softened crystal contacts in the successful TR-SSX study.^25^

Figures 2(c) and 2(d) illustrate the qualitative agreement between the Cα displacements across the set of 100 structures modeled using partial occupancy refinement or refinement against extrapolated data. This apparent agreement can be put on more quantitative footing by calculating the average Cα displacement for each of the two sets of 100 refined structures and plotting these with their corresponding error bars [Fig. 2(e)]. Moreover, when the mean coordinates are calculated from these two approaches to structural refinement, and their discrepancy is compared against the sum of their corresponding coordinate errors, it is apparent that they differ by less than one standard deviation [Fig. 2(f)]. Another perspective is offered by calculating the Pearson correlation coefficient between the two approaches to structural refinement, which yields a correlation coefficient of 70%. Although this value is lower than the mean correlation coefficient within the set of 100 structures refined using the same approach (86% when using partial occupancy refinement and 83% when using extrapolated data refinement), this is still a high level of correlation. Thus, we are able to state with confidence that, in the case of light-driven conformational changes associated with bR for Δ*t* = 1.725 ms after photoactivation, structural modeling using partial occupancy refinement and against extrapolated data using XTRAPOL8,^44^ the modeled Cα displacements agree within the estimated coordinate errors.

### Resampling difference Fourier electron density maps

Since Henderson and Moffat's influential paper on the method,^39^ isomorphous difference Fourier electron density maps have been utilized as a core tool for interpreting the outcome of a TR-XRD experiment. Two critical features are their sensitivity to small changes and the absence of model bias toward the excited state conformation. Moreover, because difference Fourier maps use the resting state conformation for phases [Eq. (1)], an isomorphous *F*_obs_ − *F*_obs_ difference Fourier electron density map most accurately represents the electron density changes when the two compared structure factors share approximately the same phase,^39^ which holds true for small and localized conformational changes but is less applicable if larger conformational changes are distributed throughout the protein. An underappreciated point of potential concern is that phase bias may arise, whereby the resting state model together with the errors in the difference amplitudes may influence the interpretation of the map. In an idealized case of zero noise, there will be no random differences in structure factor amplitudes and therefore no random contribution to the difference Fourier map. However, two sets of experimental data are never identical. Whereas scaling attempts to account for known variations between datasets, scaling protocols fall short of addressing all systematic errors, including both experimental errors and those stemming from data reduction models. Machine learning tools may offer an empirical approach to side-step many systematic errors,^61^ yet the awareness of phase bias in *F*_obs_ − *F*_obs_ isomorphous difference Fourier maps is limited and tools for its mitigation are underdeveloped. Irrespectively, there is always a subjective element associated with the interpretation of a difference Fourier electron density map, especially when there may be more than one activated-state conformation present in the data. For example, pairs of positive and negative electron density peaks are often interpreted as arising from atomic movements and are subsequently modeled as such, even when the occupancy may be low and supporting electron density may not be visible in 2*F*_obs_ − *F*_calc_ maps.

Resampling of TR-SFX data provides an avenue with which to examine the question of phase bias in difference Fourier map, since a set of resampled observations and their refined resting state structures can be utilized to calculate a set of isomorphous difference Fourier electron density maps while allowing considerable variation in the resting state phases. Thus, from 100 resamplings of the photoactivated dataset, and 100 resamplings of the resting state dataset, we calculate 100 *F*_obs_ − *F*_obs_ isomorphous difference Fourier electron density maps using Eq. (1), each with slightly different phases due to variation in the resampled resting state structures [Fig. 2(a)]. Because only a subset of the experimental data were used when calculating each of these 100 difference Fourier maps, and because the signal to noise in serial crystallography improves as more data are included, the quality of each individual resampled difference Fourier map is inferior to that calculated when using all data. Moffat and co-workers have utilized singular value decomposition to extract the time-dependence of signals within TR-XRD data and thereby improve signal to noise ratios in difference electron density maps.^40,47^ We have also implemented a similar algorithm in a TR-SFX study of ultrafast conformational changes in a photosynthetic reaction center.^30^ We again utilize this approach to analyze a set of 100 isomorphous difference Fourier electron density maps calculated from resampled data using the program PHENIX,^45^ by extracting the principal SVD component from this set. Figures 3(a), 3(c), and 3(e) present the principal SVD component resulting from this analysis for the time delays Δt = 16 ns, 760 ns, and 1.725 ms. For benchmarking purpose, this map is compared with the difference Fourier map using identical protocols to merge data and calculate the isomorphous difference Fourier map, but instead using all of the dark and light data without resampling [Figs. 3(b), 3(d), and 3(f)]. Overall, these maps are very similar, with Pearson correlation coefficients of 75% for Δ*t* = 16 ns, 87% for Δ*t* = 760 ns, and 89% for Δt = 1.725 ms. Whereas all three Pearson correlation coefficients indicate very similar quality difference Fourier electron density maps, the higher correlation coefficient calculated for the two longer time-delays presumably arises because more extensive electron density changes are visible for these time points and consequently the overall signal to noise ratio of the map is better. Because the principal SVD component of 100 resampled difference Fourier maps is very close to the corresponding difference Fourier maps calculated using more conventional approaches, it may be concluded that phase bias associated with the resting state model [Eq. (1)] does not unduly impact the final map when the experimental data has a good signal to noise ratio.

Closer inspection reveals that some details of the SVD analysis of a set of resampled difference Fourier maps may be visualized with improved confidence than for a more standard difference Fourier map. This is reminiscent of similar benefits associated with Bayesian statistics based approaches developed to improve the quality of difference Fourier electron density maps.^44,48^ More specifically, the original TR-SFX study of time-dependent electron density changes in bR emphasized how the transient disordering and ordering of water molecules^22^ was captured in a sequence of 13 time-delays from 16 ns to 1.725 ms. It has long been known that the disordering and reordering of water molecules is critical to the function of bR,^51,62,63^ since water molecules provide pathways along which protons move. One functionally important water molecule that transiently ordered was assigned as Wat452, which bridges from the protonated Schiff base (the primary proton donor) in the 13-*cis* retinal conformation of the photoactivated state, to Asp85 (the primary proton acceptor) via Thr89 [Fig. 3(c)]. For the time-delay Δ*t* = 760 ns, the positive difference electron density associated with Wat452 is better defined in the principal SVD component difference Fourier map (maximum value of +4.0σ, where σ is the root mean square electron density of the map) than in the control difference Fourier map calculated when all data were merged without resampling (maximum value of +3.5σ). The time-delays Δ*t* = 760 ns to 13.8 *μ*s are associated with the L-intermediate, which sets the scene for the primary proton transfer from the Schiff base to Asp85. Several low-temperature intermediate trapping x-ray diffraction studies of the L-state of bR have been reported,^60^ yet only one study presented evidence for a water molecule at the position of Wat452.^63,64^ Thus, the above-mentioned SVD analysis of difference Fourier maps calculated from resampled TR-SFX data, illustrates how this approach may improve confidence in the assignment of a water molecule that transiently orders, so as to assist the primary proton transfer of a proton from the Schiff base to Asp85. Similarly, positive difference electron density is associated with Wat453, which orders near the carbonyl oxygen of Ala216 and is stronger in the principal SVD component map [Fig. 3(c), maximum value of 4.1σ] calculated from data collected with Δ*t* = 760 ns than the corresponding feature in the difference Fourier map calculated without resampling [Fig. 3(d), maximum value of 2.9σ]. An improvement in the strength of positive difference density associated with Wat452 also holds for the time-delay Δ*t* = 16 ns [maximum value of 3.3σ, Fig. 3(a), vs 2.7σ], yet this is only marginally true for Δ*t* = 1.725 ms, where this density is weak in both cases (maximum values of 2.4σ vs 2.2σ, respectively).

### Error bars associated with difference Fourier and Polder omit maps

For many biological macromolecules, the transient ordering and disordering of water molecules or other ligands is central to their evolved functional mechanism. The above-mentioned analysis illustrates how SVD tools in combination with resampling techniques, allow the constraints of a single resting state model for crystallographic phases to be relaxed and this may improve confidence when assigning time-dependent difference electron density features associated with transiently ordered water molecules. When describing transient water molecules, it is common to measure and quote the maximum sigma value in a difference density map.^22,23,27,65^ A more quantitative approach is to integrate difference electron density within a sphere of defined radius about a specified atom.^46^ When utilizing this approach in combination with resampling strategies used in Resampling difference Fourier electron density maps section, it is possible to utilize the standard deviation across a set of 100 resampled difference Fourier electron density maps in order to estimate the inherent noise associated with the calculation of these maps from TR-SFX data.

**FIG. 4.**
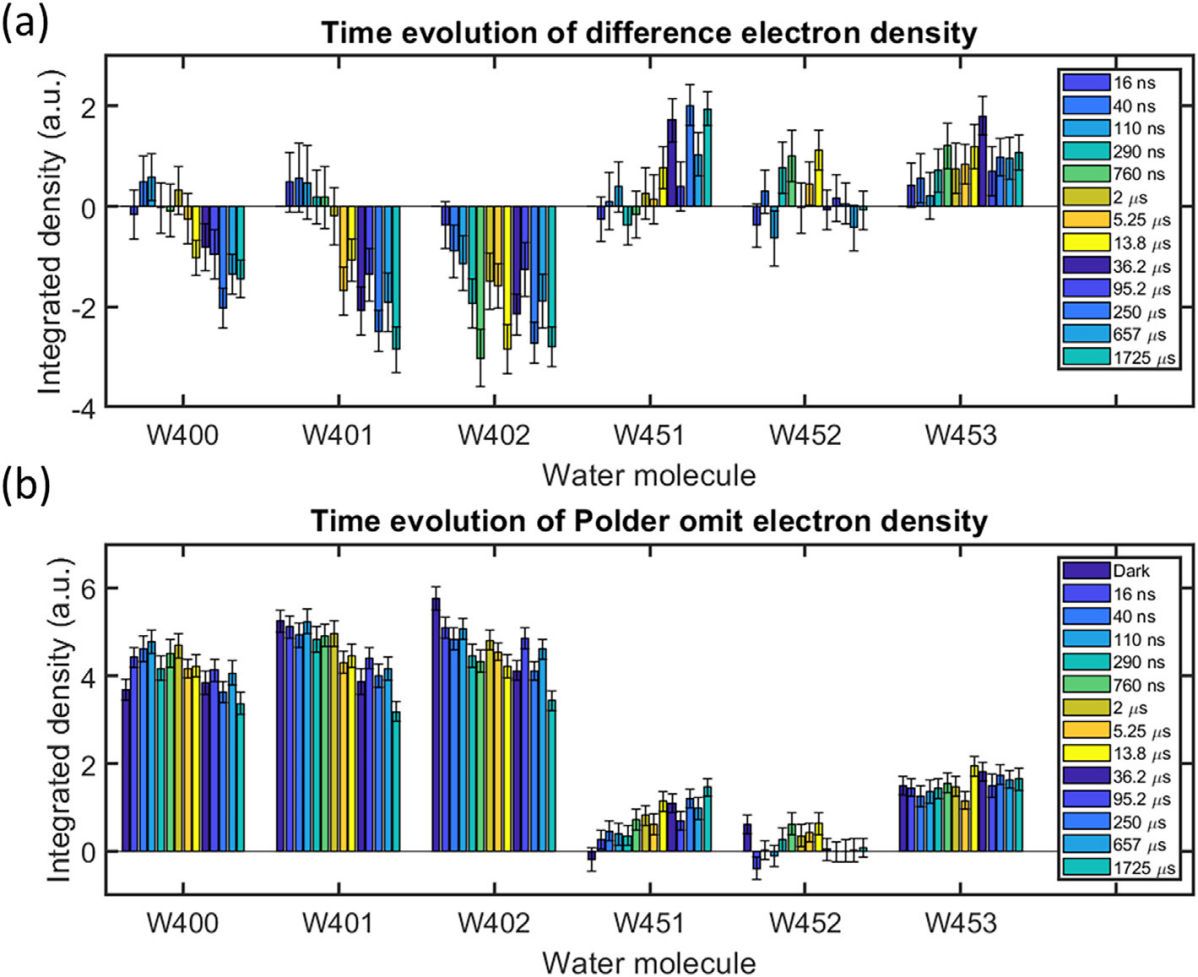
Time evolution of electron density map features associated with functional water molecules. (a) Time evolution of isomorphous *F*_obs_(light) − *F*_obs_(dark) difference Fourier electron density features associated with functional water molecules, quantified by integrating the electron density within a sphere of radius 0.8 Å about each named water molecule^46^ and setting difference electron density with an amplitude below a pedestal of ±2.0σ to 0. Error bars are given as the standard deviation of quantified values over a set of 100 resampled difference Fourier electron density maps. (c) Time-evolution of Polder omit electron density^66^ features associated with functional water molecules. These values are quantified by integrating the electron density within a sphere of radius 0.8 Å about each named water molecule and setting difference electron density with an amplitude below a pedestal of ±0.1σ to 0. Trends observed in panel (a) are reproduced in panel (b). Integrated Polder omit electron density associated with the dark state observations is also shown in panel (b).

Figure 4(a) shows the results of integrating the difference electron density within a sphere of 0.8 Å radius about six selected functionally important water molecules immediately to the extracellular [Wat400, Wat401, Wat402, and Wat451, Fig. 3(a)] and cytoplasmic [Wat452 and Wat453 Figs. 3(a) and 3(b)] sides of the retinal, for all thirteen time-delays of the earlier TR-SFX study.^22^ This reveals that the disordering of Wat402, a keystone water molecule of the resting conformation that is in hydrogen bond contact with the retinal Schiff base, begins to disorder in response to retinal isomerization by Δt = 16 ns and has reached a maximum negative density by Δt = 290 ns. In contrast, the disordering of Wat400 (Δt ≥ 13.8 *μ*s) and Wat451 (Δt ≥ 5.5 *μ*s) is somewhat delayed, and a new water (Wat451) orders between the positions of these three resting state water molecules on the same timescale (Δt ≥ 13.8 *μ*s). On the cytoplasmic side of the protein, positive difference electron density for Wat452 is only transient (strongest for Δt = 290 ns, 760 ns, and 13.8 *μ*s), whereas that associated with Wat453 appears to grow steadily throughout this study.

Complementary to this analysis of a set of 100 resampled isomorphous difference Fourier electron density maps, a Polder omit map^66^ can be used to quantify the absolute, rather than difference, electron density features associated with water molecules. As with any other omit map, Polder omit maps calculate the *F*^obs^ − *F*^calc^ electron density after removing an atom of interest from the structural model used to generate *F*^calc^ amplitudes. Polder omit maps were developed because most modern structural refinement packages compensate for bulk-solvent effects by postulating a constant electron density at any point within the unit cell that is not occupied by an atom. A Polder omit map prevents the bulk-solvent correction from entering the region near the selected atom, and therefore, weak electron density features should not be swamped by the bulk-solvent correction.^66^ In Dutch, polder refers to low-land that is reclaimed by preventing the sea from flooding the land, and by analogy, a Polder omit map tries to avoid weak electron density features being swamped by the bulk solvent.

Figure 4(b) quantifies the results of calculating Polder omit maps for the above-mentioned selected water molecules from 100 sets of resampled data, and integrating the resulting electron density within a sphere of 0.8 Å radius, for the resting conformation and all thirteen time-delays of the original TR-SFX study. Structural information revealed by this Polder omit map quantification [Fig. 4(b)] is complementary to that contained within the parallel analysis of the isomorphous difference Fourier maps [Fig. 4(a)]. For example, the loss of electron density associated with Wat400, Wat401, and Wat402 can also be read from the Polder omit analysis, as can the more rapid disordering of Wat402 be inferred from its more rapid loss of Polder omit map density. Moreover, a steady growth of electron density for Wat451 can be seen, as can electron density for Wat453 be quantified for all time delays as well as for the resting conformation. Much weaker Polder omit map electron density features are associated with Wat452, but when they arise there is an obvious correlation with the presence of positive difference electron density features in the difference Fourier analysis. However, interpreting very weak signals in Polder omit maps risks interpreting noise in the electron density map, and a warning is implied in the presence of residual Polder omit map density for Wat453 in the resting conformation [Fig. 4(b)]. As such, we suggest that the quantification of Polder omit maps from resampled TR-SFX data may assist in the interpretation of transiently changes in electron density, but there are risks when these features are very weak. We therefore recommend that any functional conclusions drawn from a similar analysis of Polder omit map electron density calculated using resampling methods, should be backed-up by transient positive difference electron density in the isomorphous difference Fourier maps, as well as functional evidence.

## CONCLUSIONS

In this work, we have utilized the resampling of TR-SFX data to explore some of the assumptions underpinning the structural interpretation of TR-XRD experiments. By working with data from an earlier successful TR-SFX experiment^22^ with good signal to noise for a time-dependent sequence of difference Fourier electron density maps, we emphasize empirical rather than theoretical foundations of the tools of structural refinement. Moreover, we explicitly build upon an inherent characteristic of serial crystallography, which allows resampling of data to simulate the results from quasi-independent measurements and thereby estimate experimental uncertainties from the spread of parameters determined from resampled data.

Figure 1 illustrates how the mean amplitude of Cα-atom displacements resulting from the structural refinement of an activated conformation varies linearly with the inverse occupancy (1/*f* ) when the occupancy is the order of 25% or more. Moreover, this finding is consistent for both partial occupancy refinement and refinement against extrapolated data.^44^ This makes explicit one of the hidden assumptions of the field, and there is value in benchmarking the assumption of linearity against empirical data so as to appreciate when this begins to breakdown. An important corollary of the linear variation of the amplitude of atomic displacements with 1/*f* is that the accuracy with which the crystallographic occupancy is estimated is a critical consideration when judging the accuracy of modeled atomic displacements. However, it is difficult to quantify the crystallographic occupancy,^37^ and there are no universally agreed protocols for this, but rather a bespoke patchwork of approaches each with their own assumptions. For the sake of consistency, here we have chosen the occupancy according to the maximum observed correlation between a set of 100 structural refinements with the mean of that set [Fig. 1(e)], but we see no reason to advocate this approach ahead of a number of other protocols that have been used in the scientific literature.

Once the crystallographic occupancy is fixed, Fig. 2 explores the variation in Cα-atom displacements from 100 resampled datasets randomly selected using bootstrapping protocols. This analysis shows that significant structure-to-structure variation emerges for the refined coordinates, and that the standard deviation in Cα coordinates is close to, but slightly larger than, an earlier simplified coordinate error estimate from single-crystal crystallography^54^ [Eq. (3)]. Nevertheless, the mean displacements of Cα-atoms for some regions of the protein (helices C, F, and G) are larger than the coordinate uncertainty bars [Fig. 2(e)], and the results from partial occupancy refinement and extrapolated data refinement are in agreement within these coordinate error estimates [Fig. 2(f)]. As with the assumption of linearity, the agreement between different structural refinement protocols within coordinate errors is another assumption of the field, and again there is value in benchmarking this hidden assumption against empirical data.

Finally, we utilize resampling to investigate another hidden assumption that phase bias toward the resting state model does not unduly impact upon the quality of the resulting difference Fourier electron density map. An SVD analysis of a set of 100 difference Fourier maps calculated from resampled data reveals that the principal component of this set is very close to the map that would be calculated using all data in a more conventional approach (Fig. 3). Nevertheless, some weaker difference electron density features associated with transiently ordered water molecules emerged with improved confidence. Moreover, this resampling allows uncertainty bars to be given for the strength of difference Fourier electron density features [Fig. 4(a)]. A similar analysis using a Polder omit map [Fig. 4(b)] allows complementary time-dependent information to be extracted from the TR-SFX dataset. These representations assist in assigning functionally important features of difference Fourier maps across a sequence of time-delays, in a manner which conveys some limitations of the experimental data.

Our reflections on some assumptions underpinning the field of TR-XRD are relevant to an ongoing debate concerning the functional relevance of crystallographic coordinates recovered from TR-XRD studies of light-sensitive proteins. More specifically, it has been noted (with one recent exception^37^) that all TR-XRD studies of light-sensitive proteins to date were performed using photoexcitation conditions in the non-linear domain.^2,67,68^ This is of concern because all light-sensitive proteins have evolved under sunlight conditions of single-photon excitation. It has therefore been argued that TR-XRD studies may describe artefactual structures that can only be populated in the artificial environment of a laser laboratory. Using the model system of photodissociation of CO from the heme iron of myoglobin, Barends *et al.*^37^ recently reported the first TR-XRD study under conditions of single-photon excitation. On a sub-picosecond timescale, the protein was observed to undergo similar ultrafast conformational rearrangements following single-photon^37^ and multiphoton excitation,^36^ yet these rearrangements are slower, and photo-dissociated CO takes more time to populate a binding-site near the active-site heme iron, when using single-photon excitation. For a time-delay of tens of picoseconds, it was not possible to distinguish between single-photon and multiphoton trajectories from the difference Fourier electron density maps, although it was suggested that subtle differences could be distinguished using structural refinement.

It is an important principle that, in order to connect with the field of time-resolved spectroscopy, TR-XRD studies must be performed under conditions of single-photon excitation.^67,69^ Our analysis illustrates how errors associated with estimating the crystallographic occupancy, atomic coordinate errors, and errors in the amplitude of transient features of difference Fourier electron density maps, all need to be considered before two trajectories can be distinguished with confidence. Moreover, these errors have a larger impact when the crystallographic occupancy very low, which is the case for single-photon excitation within protein crystals.^37,68^ If the crystallographic coordinates resulting from of single-photon and multiphoton excitation conditions are indistinguishable given these known sources of error, we suggest that there is room for pragmatism in this debate.^69^ It will be possible to perform TR-SFX studies in the single-photon regime for a small number of proteins with favorable photoexcitation properties, which produce very well diffracting microcrystals, but these requirements will not hold true for most systems of scientific interest. Nevertheless, we feel that structural results deriving from conditions of multiphoton excitation have merit as long as the major structural findings are consistent with complementary evidence from functional studies.

## Data Availability

The data that support the findings of this study are openly available in GitHub at https://github.com/Neutze-lab/resampling, Ref. 70.
